# Neighbourhood walkability and mental health in older adults: A cross-sectional analysis from EpiFloripa Aging Study

**DOI:** 10.3389/fragi.2022.915292

**Published:** 2022-11-29

**Authors:** Joel de Almeida Siqueira Junior, Adalberto Aparecido dos Santos Lopes, Carla Elane Silva Godtsfriedt, Marcelo Dutra Della Justina, Karina Mary de Paiva, Eleonora d’Orsi, Cassiano Ricardo Rech

**Affiliations:** ^1^ Center of Sports, Federal University of Santa Catarina, Florianópolis, Santa Catarina, Brazil; ^2^ Observatory of Urban Health, Federal University of Minas Gerais, Belo Horizonte, Minas Gerais, Brazil; ^3^ Department of Speech Therapy, Federal University of Santa Catarina, Florianópolis, Santa Catarina, Brazil; ^4^ Department of Public Health, Federal University of Santa Catarina, Florianópolis, Santa Catarina, Brazil

**Keywords:** walkability index, cognitive impairment, depressive symptoms, physical activity, older adults

## Abstract

This study aims to analyse the association between walkability index and depressive symptoms and cognitive impairment and test the mediating role of moderate-vigorous physical activity (MVPA) in this relationship among older adults from Florianópolis, Brazil. This is cross-sectional research with data from the third wave of the EpiFloripa Aging cohort study, conducted in 2017–2019. Depressive symptoms were assessed using the short version of the Geriatric Depression Scale (GDS), and cognitive impairment, using the Mini-Mental State Examination (MMSE) scales. The neighbourhood environment was assessed using a walkability index, which considered 500-m network buffers around the participants’ homes. Binary logistic regression analysis the association between the walkability index (quartile) and mental health outcomes (yes vs*.* no). Structural equation modelling evaluated the mediation between the walkability index and cognitive impairment by MVPA with an estimator of dichotomous variables. 1,162 people participated in the study (61.5% women, average age = 73.1). Older adults residing in places with a high and highest walkability index were 38% and 44% less likely to have cognitive impairment, respective. There was no association between depressive symptoms and walkability index in crude nor adjusted analysis. Engaging in MVPA had a partial but not significant effect (14%; *p* = 0.087), showing a tendency for this relationship to be partially explained by the greater engagement in physical activities in places with greater walkability. Policy planning to prevent and reduce the risks of cognitive impairment should consider factors of the physical environment as determinants in older adults.

## 1 Introduction

In recent years, mental disorders have increased among older adults and represent a significant risk factor for loss of functional independence and longevity. It also has a personal impact, such as loss of functional capacity, family dependency and can affect healthcare systems (Giebel et al., 2016). Thus, the ability to age with preserved mental health has been the focus of attention in recent decades (Billal et al., 2021). The prevalence of depressive symptoms in older adults (≥60 years) has increased considerably around the world in recent years, ranging from 16% to 22% (Ismail et al., 2017), and the projection for 2050 is for an increase from 57.4% to 117% in the prevalence of cognitive impairment, affecting up to 152 million older adults (Nichols et al., 2019). These numbers can be even more worrying in low- and middle-income countries due to precarious access to health care, low level of education, many people residing in urban areas with poor infrastructure, and low social support (Li & Shou, 2021). Prospects also evidence a synergy between diseases, in the sense that the incidence of depressive symptoms can increase the risk of progression to cognitive impairment by two to five times (Mussele et al., 2014).

Despite the morbidities and increased chances of mortality due to a combination of health risk factors in the older adults, it is possible to compensate or decrease these risk factors by adopting healthier habits. The relationship between physical activity and mental health conditions has been well established (Hanson & Jones, 2015). Regular physical activity can promote cognitive development, delay symptoms of dementia, and prevent and treat depressive symptoms in older adults (Chen et al., 2017). Walking stood out, being the most prevalent activity in this population and considered a simple, low-cost strategy that makes them physically active and reduces mental health issues (Bonaccorsi et al., 2020). Therefore, providing better spaces for leisure-time walking has become one of the biggest challenges in global government agendas to boost care actions aimed at the mental health of older adults (Roe et al., 2020).

The physical and organizational structure of neighbourhoods can influence the mental health of senior citizens, either through their perception of aesthetics, safety and accessibility conditions or even through the geographic variations of the built environment (Melis et al., 2015; Wu et al., 2015). Thus, considering the characteristics of the neighbourhood environment in the formulation and implementation of public policies has presented significant evidence that makes it possible to encourage walking among older adults (Paiva Neto et al., 2021). The walkability, as an index of a neighbourhood’s ability to support walking in people’s daily lives (Frank et al., 2021), can be a means to monitor cities to create urban spaces that increase engagement in healthy behaviours (Mayne et al., 2018). Possible to measure using geospatial data, this index is composed of a set of indicators, such as residential density, the intersection of streets, mixed land use, and retail area (Frank et al., 2010), providing a better understanding of the urban environment, due to the complexity of factors that act in the same period in the daily life of citizens. A walkable neighbourhood can be better explored by the population, stimulating local commerce, social interactions among neighbours, and softening the impacts of depressive symptoms and cognitive decline in the older population (Gong et al., 2016).

From this perspective, the characteristics of the neighbourhood environment, such as mixed land use, availability of green areas and street connectivity, stimulate high levels of walkability (Watts et al., 2015; Wu et al., 2017). In the opposite direction, insecurity, inaccessible or poorly maintained sidewalks, and disruptions in the surrounding environment can impair cognitive performance in older adults (Cassarino & Setti, 2015). Also, in this context leisure-time physical activity can play an essential mediating role and explain the relationship between walkability and the cognitive, affective and social processes that justify aspects of mental health in urban centres (Solis-Urra et al., 2020).

After considering the characteristics of the neighbourhood environment as a potential for increasing neighbourhood walkability by older adults, this study hypothesizes that those older adults who reside in neighbourhoods with a higher walkability index are less likely to develop cognitive impairment and that moderate to vigorous leisure-time physical activity can mediate this relationship. Thus, the objective of this study was to analyse the association that the walkability index has with depressive symptoms and cognitive impairment, as well as to test the mediating role of moderate-vigorous physical activity (MVPA) in this relationship among older adults from Florianópolis, Brazil.

## 2 Materials and methods

### 2.1 The site, study design and ethical procedures

This is population-based, cross-sectional research using data from the third wave of the “EpiFloripa Aging: Living Conditions and Health of Older Adults from Florianópolis” cohort study, conducted between October 2017 and December 2019. The capital of the state of Santa Catarina had a population of 421,240 inhabitants, according to the last census of the Brazilian Institute of Geography and Statistics in 2010 (IBGE, 2011), and a territorial area of 675,409 km^2^ divided into 651 census sectors—603 urban and 48 rural. The city also had a Municipal Human Development Index (HDI-M) of 0.847, with the third best among Brazilian municipalities (UNDP, 2013).

### 2.2 Population and sample selection

The study involved older adults (≥60 years) living in the urban area of Florianópolis, Brazil. The third wave of the research became an open-cohort study with the inclusion of new older adults in the sample and those who participated in the previous waves and were being followed up. It started in 2017 and ended in 2019 with 1,335 older adults interviewed. To set the sample size, the formula for calculating the unknown prevalence (50%) was used, based on the reference more ageing population of 44,460 inhabitants, through a simple casual sample (confidence level of 1.96 standard deviations, 4% sampling error.

From identifying the profile of the older adults eligible to compose the Wave 3 sample of the EpiFloripa Aging study and maintaining an average of 20 older adults per sector, it was identified that between 3 and 16 older adults should be interviewed in each census sector. In this way, the average number of people per household (421,240 inhabitants/147,406 homes) and the proportion of older adults in the municipality (11.4%) were considered, resulting in an average of one older adult for every three households, with this formula being used: number of new older adults needed in the sector, with an increase of 20% for expected losses, multiplied by three (number of older adults in each household). Institutionalized older adults were considered as an exclusion criterion. Previous publications present the details of the study sample and methodology (Schneider et al., 2017; Confortim et al., 2019).

### 2.3 Outcomes

The presence of depressive symptoms was assessed using the short version of the Geriatric Depression Scale (GDS), a structured questionnaire ranging from 15-item (no/yes response options), which has a score ranging from 0 (absence of depressive symptoms) to 15 points (maximum score for depressive symptoms). For analytical purposes, the cut-off point of >6 was considered indicative of depressive symptoms in older adults (Almeida and Almeida, 1999).

Cognitive impairment was assessed using the Mini-Mental State Examination (MMSE) questionnaire, ranging from 0 to 30 points. The MMSE is a cognitive assessment test that analyzes different cognitive domains, such as time perception, spatial orientation, short-term memory, calculation, comprehension and writing (Folstein et al., 1975). In this study, the cut-off points used were that probable cognitive decline is indicated by a score of <19 points for individuals with no education and <24 points for those with some level of education (Almeida, 1998). For data analysis, the older adults were classified into “absence” and “presence” of cognitive impairment.

### 2.4 Exposure

For the 1,335 participants in Wave 3 of the EpiFloripa Aging cohort study, it was possible to georeference 1,162 older adults who had complete address data. Then, 500-m network buffers were created around each participant’s residence (Frank et al., 2017). The walkability index was built using the ArcGIS software, version 10.5, owned by ESRI^®^, and taking into account three indicators: residential density, obtained by the ratio of the total number of residences divided by the total area of the buffer in Km^2^; the intersection of streets, estimated from the balance between the number of junctions formed by three or more segments, divided by the total area of the buffer in Km^2^; and mixed land use, obtained by calculating the entropy, which varies from zero (only one type of land use) to one (equal distribution among all land use categories) (Frank et al., 2010). For analysis purposes, the quartile values of the walkability index were considered and categorized as lowest (<−2.16), low (−2.17; −0.97), high (−0.98; 0.75) and highest (>0.76). Details on the walkability index development can be accessed in the preview publication (Justina, 2021).

### 2.5 Covariates

Sociodemographic information was used as control variables, with sex (female and male) being obtained through observation by the researcher. Age was obtained through the participant’s date of birth, computed along with the date of their respective interview and classified into three categories—“60–69 years old,” “70–79 years old,” and “80 years old” or more. Marital status was obtained and classified as “married”, “single”, “divorced” and “widowed”. Education was collected in complete years of study and classified into three categories—“≤4 years,” “5–8 years,” and “≥9 years”. The percentage of older adults with a monthly income equal to or less than one minimum wage was calculated in accordance with the current value of the per capita income, between R$1,387.00 and R$1,406.00, in the year of the interview.

Alcohol consumption was obtained through the “The Alcohol Use Disorders Identification Test-Concise” (AUDIT-C) questionnaire (Babor et al., 2001) and categorized as “never,” “moderate use,” and “high”. Tobacco consumption was defined by the affirmative answer and classified as “never”, “smoked and quit”, and “currently smokes”. Body Mass Index (BMI) was calculated by dividing weight by height squared and classified as underweight (<22 kg/m2), normal weight (22–27 kg/m2) and overweight (>27 kg/m2) (Lipschitz, 1994). To estimate self-perception of health, the “regular”, “poor”, and “very poor” categories were classified as negative self-perception of health, while “very good” and “good” were grouped and classified as positive self-perception of health (Ware Junior, 1996). Moderate and vigorous physical activity (MVPA) in the leisure domain was assessed using the International Physical Activity Questionnaire (IPAQ) extended version, which was validated to be used with older people in Brazil (Benedetti et al., 2004); based on weekly frequency and length in minutes, it was categorized as insufficiently active (0–149 min/wk) and active (>150 min/wk).

### 2.6 Data analysis

The categorical variables were described utilizing their frequency distributions (absolute and relative), and the quantitative variables, by the central tendency and dispersion measures (mean and standard error). The association between the walkability index and the mental health of the older adults was tested using Binary Logistic Regression, considering the dichotomous character of the outcomes (depressive symptoms and cognitive impairment) and the non-normality of the exposure (walkability index), which was classified as lowest, low, high and highest walkability, by the quartile values. In addition to the crude models, adjusted models were tested, which considered the sociodemographic profile and health behaviours associated with the outcomes in the crude analysis. The binary logistic regression analyses were run on the SPSS^®^ statistical software, version 21.0.

The model for mediation of physical activity in cognitive decline was assessed with the aid of the Mplus 13.1 software, using the WLSMV (Mean and Variance-Adjusted Weighted Square) method applied to the original items. The model’s goodness of fit analysis used the CFI (Comparative fit index) and GFI (Goodness-of-fit index), which were considered to indicate a good fit for values greater than 0.90. The RMSEA (Root mean square error of approximation) was also used, with 90%CI with an upper limit lower than 0.05 as a good fit (Maroco, 2010). The mediation model was only tested for cognitive decline, as it met the following conditions: 1) the exposure variable (walkability) was significantly associated with the outcome (depressive symptoms or cognitive impairment); 2) the exposure variable (walkability) was significantly associated with the mediator (MVPA); 3) the mediating variable (MVPA) was significantly associated with the outcome variable, and; 4) the exposure variable had its magnitude of association with the outcome reduced after the insertion of the mediating variable (Baron & Kenny, 1986). For all analyses, a significance of 5% was adopted.

## 3 Results

A total of 1,335 older adults participated in the study, 173 of which (12.9%) were considered losses due to non-response in any outcome or exposure variables. The analysis included 1,162 people (61.5% women), aged 60 years (mean = 73.1; standard deviation = 7.82). Most of the sample was between 70 and 79 years old (41.4%), were married (56.5%), had more than 9 years of education (46.9%), and 90.7% had an income of up to one minimum wage. In the sample, 57% reported not consuming alcohol, 58.7% did not smoke, 54.6% were overweight, 62.6% reported a positive perception of health, and 88.1% were inactive. As for mental health, 14.7% had depressive symptoms, and 16.8% had cognitive impairment (Table 1).

**TABLE 1:** Characteristics of the participants of the EpiFloripa Aging Study. Florianopolis, Brazil, 2017–2019 (n = 1,162).

Variables	n	% (CI_95%_)
Sex		
Female	715	61.5 (58.7–64.3)
Male	447	38.5 (35.7–41.3)
Age group (in years)		
60 to 69	420	36.1 (33.4–38.9)
70 to 79	481	41.4 (38.6–44.2)
≥80	261	22.5 (20.1–24.9)
Marital status		
Married	657	56.5 (53.7–59.4)
Single	77	6.6 (5.3–8.2)
Divorced	136	11.7 (10.0–13.7)
Widower	292	25.1 (22.7–27.7)
Education level (in years)		
≤4 years	404	34.8 (32.0–37.6)
5–8 years	213	18.3 (16.2–20.7)
≥9 years	545	46.9 (44.0–49.8)
Income		
≤1 minimum salary	1042	89.7 (88.9–92.2)
>1 minimum salary	107	9.2 (7.8–11.1)
Missing	13	1.1
Consumption of alcoholic		
Never	662	57.0 (54.0–59.8)
Moderate	254	21.9 (19.6–24.3)
High	246	21.1 (18.9–23.6)
Smoking habit		
Never	682	58.7 (55.8–61.5)
Smoked and stopped	372	32.0 (29.4–34.8)
Smoke	108	9.3 (7.8–11.1)
Body Mass Index*		
Low Weight	106	9.3 (33.3–38.9)
Eutrofic	411	36.1 (7.7–11.1)
Overweight	622	54.6 (51.7–57.5)
Missing	23	2.0
Self-rated health		
Negative	435	37.4 (34.7–40.3)
Positive	727	62.6 (59.7–65.3)
Moderate-vigorous physical activity		
Inactive	1019	87.7 (86.0–89.9)
Active	138	11.9 (10.1–13.9)
Missing	5	0.4
Depressive Symptoms**		
Normal	991	85.3 (83.1–87.2)
Suspicion of depression	171	14.7 (12.7–16.8)
Cognitive impairment ***		
Absence	967	83.2 (80.9–85.2)
Presence	195	16.8 (14.7–19.0)
Walkability Index		
Lowest	294	25.3 (22.8–27.8)
Low	293	25.2 (22.7–27.7)
High	287	24.7 (22.3–27.2)
Highest	288	24.8 (22.4–27.3)

MVPA: Inactive (<150 min/week), Active (>150 min/week); *: BMI Cutoff point (LIPSCHITZ, 1994) (< 22 kg/m2, low weight; >22 e <27 kg/m2: eutrophic; > 27 kg/m2: overweight); **: Depressive symptoms based on Geriatric Depression Scale; ***: Cognitive impairment examined by Mini Mental State Examination.

The crude analysis showed that older adults aged 80 years and older (OR: 1.62; 95%CI: 1.07–2.45) have a higher chance of having depressive symptoms, on the other hand those older adults with a higher level of education (OR: 0.33; 95%CI: 0.23–0.49), who consume alcohol in moderate amounts (OR: 0.52; 95%CI: 0.34–0.81) and in high amounts (OR: 0.29; 95%CI: 0.17–0.50) are less likely to have depressive symptoms. Regarding cognitive impairment, those with a higher level of education (OR = 0.06; 95%CI: 0.03–0.10), who consume alcohol in moderate amounts (OR: 0.34; 95%CI: 0 0.21–0.54) and high amounts (OR:0.46; 95%CI: 0.30–0.71) are less likely to have this outcome. On the other hand, those who are older (≥80 years) (OR: 3.83; 95%CI: 2.53–5.81) and who are widowed (OR: 2.74; 95%CI: 1.94–3.86) are more likely to go through a cognitive decline (Table 2).

**TABLE 2:** Crude association between descriptive characteristics and mental health from older adults in Florianopolis, Brazil. EpiFloripa Aging Study, 2017–2019 (n = 1,162).

Indicator	Depressive symptoms			Cognitive impairment		
	n (%)	OR (CI95%)	p-value	n (%)	OR (CI95%)	p-value
All	171 (14.7)	(0.13–0.17)		195 (16.8)	(0.15–0.19)	
Sex						
Male	55 (12.3)	1	0.067	69 (15.4)	1	0.332
Female	116 (16.2)	1.38 (0.98–1.95)		126 (17.6)	1.17 (0.85–1.62)	
Age Group (in years)						
60 to 69	56 (13.3)	1		42 (10.0)	1	
70 to 79	63 (13.1)	0.98 (0.67–1.44)	0.917	75 (15.6)	1.66 (1.11–2.49)	0.013
≥80 years	52 (19.9)	1.62 (1.07–2.45)	0.023	78 (29.9)	3.84 (2.53–5.81)	< 0.001
Marital Status						
Married	86 (13.1)	1		82 (12.5)	1	
Single	13 (16.9)	1.35 (0.71–2.55)	0.358	10 (13.0)	1.05 (0.52–2.12)	0.899
Divorced	18 (13.2)	1.01 (0.59–1.75)	0.964	21 (15.4)	1.28 (0.76–2.15)	0.351
Widower	54 (18.5)	1.51 (1.04–2.19)	0.031	82 (28.1)	2.74 (1.94–3.86)	< 0.001
Education level (in years)						
≤4 years	89 (22.0)	1		146 (36.1)	1	
5–8 years	35 (16.4)	0.70 (0.45–1.07)	0.100	32 (15.0)	0.31 (0.20–0.48)	< 0.001
≥9 years	47 (8.6)	0.33 (0.23–0.49)	< 0.001	17 (3.1)	0.06 (0.03–0.10)	< 0.001
Income						
≥1 minimum salary	17 (15.9)	1		28 (26.2)	1	
≤1 minimum salary	149 (14.3)	0.88 (0.51–1.53)	0.656	163 (15.6)	0.52 (0.33–0.83)	0.006
Consumption of alcoholic						
Never	127 (19.2)	1		145 (21.9)	1	
Moderate	28 (11.0)	0.52 (0.34–0.81)	0.004	22 (8.7)	0.34 (0.21–0.54)	< 0.001
High	16 (6.5)	0.29 (0.17–0.50)	< 0.001	28 (11.4)	0.46 (0.30–0.71)	< 0.001
Smoking habit						
Never	110 (16.1)	1		123 (18.0)	1	
Smoked and stopped	44 (11.8)	0.70 (0.48–1.02)	0.060	56 (15.1)	0.81 (0.57–1.14)	0.219
Smoke	17 (15.7)	0.97 (0.56–1.70)	0.919	16 (14.8)	0.79 (0.45–1.39)	0.415
Body index mass						
Eutrophic	63 (15.3)	1		85 (13.7)	1	
Low Weight	19 (17.9)	1.21 (0.69–2.12)	0.515	75 (18.2)	1.69 (1.03–2.77)	0,038
Overweight	89 (14.3)	0.92 (0.65–1.31)	0.651	29 (27.4)	0.71 (0.51–1.00)	0.047
Self-hated health						
Positive	40 (5.5)	1		93 (12.8)	1	
Negative	131 (30.1)	7.40 (5.07–10.8)	<0.001	102 (23.4)	2.08 (1.53–2.85)	< 0.001
Walkability Index						
Lowest	49 (16.7)	1		77 (26.2)	1	
Low	41 (14.0)	0.81 (0.52–1.28)	0.369	46 (15.7)	0.53 (0.35–0.79)	0.002
High	40 (13.9)	0.83 (0.53–1.31)	0.428	40 (13.9)	0.46 (0.30–0.70)	< 0.001
Highest	171 (14.7)	0.81 (0.51–1.27)	0.352	32 (11.1)	0.35 (0.23–0.55)	< 0.001
						

MVPA: Insufficiently active (<150 min/week), Active (≥150 min/week); *: BMI Cutoff point (LIPSCHITZ, 1994) (< 22 kg/m^2^, low weight; ≥22 e < 27 kg/m^2^: eutrofic; ≥27 kg/m^2^: overweight); **: Depressive symptoms based on Geriatric Depression Scale; ***: Cognitive impairment examined by Mini Mental State Examination.

There was an association in both crude and adjusted analysis between walkability and cognition. The adjusted analysis shows that older adults residing in places with a high and highest walkability index were 38% OR = 0.62; 95%CI: 0.38–0.99) and 44% (OR = 0.56; 95%CI: 0.34–0.92) less likely to experience cognitive impairment. There was no association between depressive symptoms and walkability index (OR = 0,96; 95%CI: 0.58–1.59) in crude nor adjusted analysis (Tables 2, 3).

**TABLE 3:** Adjusted association between neighborhood walkability and mental health in older adults from Florianopolis, Brazil. EpiFloripa Aging Study, 2017–2019 (n = 1,162).

Walkability index	Depressive symptoms^a^ (GDS)		Cognitive impairment^b^ (MMSE)	
	OR (CI_95%_)	*p*-value	OR (CI_95%_)	*p*-value
Lowest	1		1	
Low	0.91 (0.56–1.50)	0.722	0.72 (0.46–1.14)	0.170
High	0.99 (0.60–1.62)	0.956	0.62 (0.38–0.99)	0.049
Highest	0.96 (0.58–1.59)	0.873	0.56 (0.34–0.92)	0.022

^a^ Adjusted model with age, marital status, education level, consumption of alcoholic and self-hated health.

^b^ Adjusteptad model with age, marital status, education level, consumption of alcoholic, BMI, and self-hated health.

Engagement in moderate and vigorous physical activity had a partial and not statistically significant effect (14%; *p* = 0.087) on the relationship between walkability and cognitive impairment (Table 4).

**TABLE 4:** Model of mediation by physical activity and association between neighborhood walkability and cognitive decline in older adults. EpiFloripa Aging Study. Florianópolis, Brazil, 2017–2019 (n = 1,162).

Potential mediator	c’-path coeficient (SD)	a-path coeficient (S.D.)	b-path coeficient (S.D.)	Indirect effect (a*b)	Percent mediation (%)	*p*-value
MVPA	−0.13* (0.04)	0.10* (0.05)	−0.21* (0.07)	−0.021	14	0.087

MVPA: moderate-vigorous physical activity. Walkability index and cognitive impairment; a, crude regression coefficient for the association between walkability index and MVPA; S_a,_ standard error of a; b, crude coefficient for the association between MVPA, and cognitive impairment; S_b,_ standard error of b; *p*-value of Structural Equations Model with MPLUS.

## 4 Discussion

The study results showed that older adults living in places with better conditions for walking, with greater street connectivity, greater residential density and greater mixed land use have a lower prevalence of cognitive impairment. Moreover, a higher level of education, being physically active and having a positive perception of health decrease the chances of experiencing depressive symptoms and cognitive impairment. These findings are essential when it comes to prioritizing urban characteristics, directing them toward active mobility through walking-friendly environments, creating and maintenance of public roads aimed at pedestrians, enabling the promotion of mental health in the population through regular engagement in physical activities in urban centres (Lee et al., 2019).

There was no association between the walkability index and the prevalence of depressive symptoms in this study. Depression is considered the most frequent mental disorder in the population over 60 years of age and represents an important risk factor for dementia and decreased functional capacity (Fiske et al., 2009). In the present study, 14.5% of the participants presented depressive symptoms, with a higher prevalence in women (16.2%) and in older adults at a more advanced age (19.9%). Even with a difference in the prevalence of depressive symptoms, this condition did not differ significantly according to the neighbourhood walkability index. This result disagrees with studies that show that senior citizens who live in places with unfavourable environmental conditions for walking are more likely to have depressive symptoms (Bolstad et al., 2020). Additionally, residing in these places could mean greater unsafety due to poorer street lighting, uneven sidewalks and heavy traffic, which can interfere with the social interaction of older adults with their neighbours, reducing social support and time for physical activity, consequently leading to an increased prevalence of depressive symptoms (Bolstad et al., 2020). The non-association between the walkability index and depressive symptoms in this study can be related to using of the macroscale of the environment (walkability), and other factors more relationship to the microscale as proximity to green areas or blue places can explain this relation in the Latin context.

Older adults living in neighbourhoods with a higher walkability index had lower cognitive impairment. Similar results have been found in the literature and confirmed that community centres, bus stops, libraries, restaurants and quality public spaces are shown to be positively associated with cognitive function (Guo et al., 2019; Finlay et al., 2020). This is possibly due to the opportunities for engaging in healthy behaviours or to a greater cognitive stimulation provided by social interaction among peers (Clarke et al., 2015). In this sense, improving public urban planning policies that provide more friendly community environments, with less pollution and noise, for older adults is imperative on government agendas (Burton, 2012). So is developing social and health promotion programs that strengthen the mental health of this population (World Health Organization, 2017), mainly because of their greater vulnerability, which can limit active commuting, leading older adults to spend more time in their residential neighbourhoods and depend primarily on the services offered, such as convenience and grocery stores (Zhang et al., 2020).

In a causal relationship, the literature suggests that people with high levels of physical activity substantially reduces the risk of developing cognitive impairment (Blondell et al., 2014). Likewise, high levels of walkability can increase physical activity by up to 57% by providing environments with a diversity of options and access to businesses and services in the neighbourhood, parks with adequate structures for use, regular and well-maintained sidewalks, and well-connected streets (Reis et al., 2013; Balcetis et al., 2020). Thus, statistical models interpreted in isolation may establish that genuine relationships between variables are to be discarded (Mc Shane et al., 2019; Wasserstein et al., 2019). Assuming statistically significant differences simply refers to accepting or rejecting a null hypothesis without providing information about its magnitude or direction (Agler & Boeck, 2017). The findings of the present study show that, although there is no statistical significance (*p* = 0.087), the indirect effect of physical activity between outcome and exposure has an important clinical and epidemiological character. Indeed, physical activity behaviours have shown a considerable mediation between characteristics of the physical environment and the mental health of older adults (Van-Dyck et al., 2015). The 14% relationship for physical activity mediation found in this study can be explained, in part, by the fact that regions with better urban design characteristics offer more options for active behaviour, which is related to more significant social interaction (Igi-Elegbede et al., 2020), thus attenuating cognitive impairment. In this way, prioritizing interventions that restructure the urban landscape, allowing greater access to green spaces close to home by means of quality sidewalks, public safety, well-maintained street furniture, seems to be a feasible strategy to enable greater engagement in physical activity and reduced levels of cognitive impairment in the course of life (Gelius et al., 2020; Igi-Elegbede et al., 2020).

The interpretation of the results must consider some limitations. The statistical analysis was based on cross-sectional data that does not allow for establishing a causal relationship between the variables; the effect of the neighbourhoods walkability on mental health may require a specific exposure time so that it is possible to identify changes in outcomes, and this was not considered; data on race/ethnicity were not used in this study and would be able to elucidate individual and sociocultural characteristics, sense of belonging and walkability in the neighborhood. However, 13.9% of the older adults reported living at the same address for at least 15 years, which may have mitigated this limitation; objective measures referring to the environment (walkability) were used, but the adoption of subjective measures could consider the value, feelings and thoughts of older adults about the neighbourhood environment; the physical activity measure was self-reported, and although a valid instrument that has been widely used in investigations with older adults was applied, it may be less precise in measuring specific intensities of physical activity, such as vigorous-intensity, when compared with accelerometer measures; the assessment of the cognitive variables and depressive symptoms was based on valid instruments, but may differ from clinical diagnosis standards; finally, the sample consisted of older adults from a city in the south of Latin America, so extrapolation to other contexts must be done with caution, as the measures of the environment may be different from those presented here.

The study’s strength is analysing a representative sample of older adults from a coastal capital. All participants were interviewed face-to-face at their homes, which can improve the quality of the information obtained. Instruments with validity were used to assess cognitive deficits and depressive symptoms in Latin America. It was possible to use the neighbourhood walkability index, an internationally accepted measure, to analyse the macroscale conditions of the place to walk. Finally, to the best of our knowledge, this is one of the first studies to deepen the discussion of the relationship between the neighbourhood environment and mental health among older adults in the Latin American context, testing the mediating role of physical activity in this association.

## 5 Conclusion

The results of this study reinforce the association observed between the neighbourhood environment and cognitive impairment in the older population and that there is a tendency that this relationship is partially explained by a greater engagement in physical activities in places with greater walkability. Policy planning for the prevention and/or reduction of dementia risks should consider improving the walkability of cities to promote the population’s mental health. The WHO has established 2020–2030 as the decade of healthy ageing, and one of the focuses is to ensure the creation of more age-friendly cities, so it is important to consider that places with lower walkability may represent possible risk factors for cognitive decline. On the other hand, encouraging physical activity by revitalizing public spaces or programs to support healthy habits can be essential to reducing the effects of low walkability on mental health. Longitudinal population-based studies are needed to clarify causal directions and investigate possible explanatory mechanisms between the built environment and mental health.

## Data Availability

The datasets presented in this study can be found in online repositories. The names of the repository/repositories and accession number(s) can be found below: https://repositorio.ufsc.br/handle/123456789/219631.
